# The Role of the PI3K Signaling Pathway in CD4^+^ T Cell Differentiation and Function

**DOI:** 10.3389/fimmu.2012.00245

**Published:** 2012-08-13

**Authors:** Jonathan M. Han, Scott J. Patterson, Megan K. Levings

**Affiliations:** ^1^Department of Surgery, Child and Family Research Institute, The University of British ColumbiaVancouver, BC, Canada

**Keywords:** CD4^+^ T cells, regulatory T cells, PI3K, AKT, FOXP3, mTOR, rapamycin

## Abstract

The relative activity of regulatory versus conventional CD4^+^ T cells ultimately maintains the delicate balance between immune tolerance and inflammation. At the molecular level, the activity of phosphatidylinositol 3-kinase (PI3K) and its downstream positive and negative regulators has a major role in controlling the balance between immune regulation and activation of different subsets of effector CD4^+^ T cells. In contrast to effector T cells which require activation of the PI3K to differentiate and mediate their effector function, regulatory T cells rely on minimal activation of this pathway to develop and maintain their characteristic phenotype, function, and metabolic state. In this review, we discuss the role of the PI3K signaling pathway in CD4^+^ T cell differentiation and function, and focus on how modulation of this pathway in T cells can alter the outcome of an immune response, ultimately tipping the balance between tolerance and inflammation.

## Introduction

Immune tolerance is a state where the immune system is unable to mount an inflammatory response toward a particular substance, thus ensuring inappropriate immunity to self or non-harmful foreign antigens is kept in check. Tolerance is controlled by two major mechanisms: central tolerance in the thymus that results in deletion of the majority of self-reactive T cells; and peripheral tolerance, which is mediated by a variety of pathways and processes including anergy, deletion, and immune regulation. Since the breakdown of immune tolerance can lead to a variety of diseases such as type 1 diabetes, allergy, and inflammatory bowel disease, there is intense research into the cellular and molecular mechanisms which control this process.

In the past 10 years, immune regulation mediated by specific types of T cells has emerged as one of the most important mechanisms of peripheral tolerance. Specifically, specialized T cells known as regulatory T cells (or T_regs_) emerge in the thymus and periphery and are dedicated to turning off immune responses (Sakaguchi et al., 2010; Rudensky, 2011). There are several different types T_regs_, including specialized subsets of CD4^+^, CD8^+^, double negative CD3^+^CD4^−^CD8^−^, γδ T cells, and NKT cells (Allan et al., 2008). While it is likely that these different types of T_regs_ work together in a network to maintain immune homeostasis, the majority of current research is focused on CD4^+^ T_regs_ since these cells are known to mediate dominant, long lasting, and transferable tolerance in experimental models (McMurchy et al., 2011).

Recently, the phosphatidylinositol 3-kinase (PI3K) signaling pathway has emerged as a key molecular regulator of immune tolerance. Modulating this pathway using drugs or genetic manipulation has revealed the importance of PI3K and its downstream signaling components in regulating T_reg_ development and function, maintaining the balance between T_regs_ and conventional CD4^+^ T cells, and controlling the distinct metabolic requirements of different CD4^+^ T cell subsets. In this review, we will discuss the current state of knowledge on how PI3K, its downstream signaling pathways, and its negative regulators, control the development and function of CD4^+^ T cells, with a specific focus on T_regs_ and immune tolerance.

## Overview of the PI3K Signaling Pathway in T Cells

**Figure 1:**
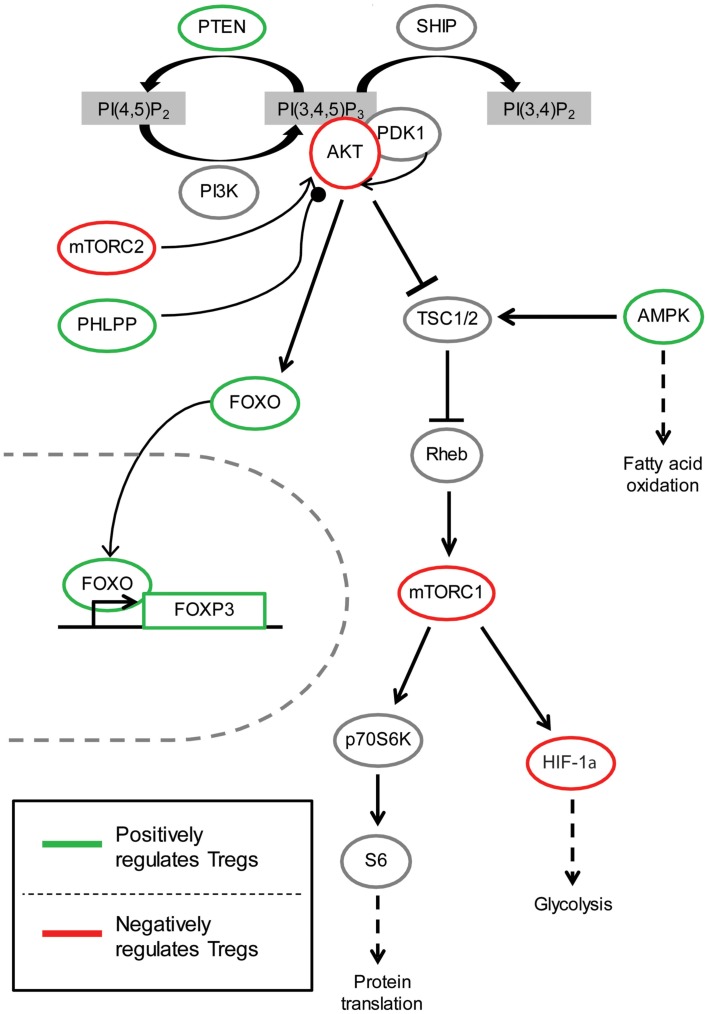
**The role of PI3K/AKT signaling pathway in T_regs_**. Arrow indicates activating phosphorylation; line with a perpendicular line at the end indicates inhibitory phosphorylation; line with a circle at the end indicates dephosphorylation; dashed line indicates resulting outcome of signaling. Green represents components of the PI3K/AKT pathway which have been shown to be beneficial for T_reg_ function and/or development. On the contrary, red indicates molecules with activity thought to be inhibitory for T_reg_ function and/or development.

Although there are four classes of PI3K, only class IA and class IB PI3K have been comprehensively studied in T cells. Most research is focused on the p110δ class IA catalytic subunit and the p110γ class IB catalytic subunit since these proteins are preferentially expressed in leukocytes (Huang and Sauer, 2010). Class IA PI3Ks are activated by receptor tyrosine kinases such as cytokine receptors and the T cell receptor (TCR), while class IB PI3Ks are primarily activated by G protein-coupled receptors (GPCRs) such as chemokine receptors. Class I PI3K phosphorylates phosphatidylinositol-4,5-bisphosphate [PI(4,5)P2] to form phosphatidylinositol-3,4,5-triphosphate [PIP3] on the inner membrane of the cell, thus initiating the recruitment and activation of downstream signaling components such as PDK1 and its substrate AKT (Figure 1). AKT activation requires phosphorylation by PDK1 at Thr308, and for full activation, and a subsequent second phosphorylation by mTORC2 or DNA-PK at Ser473 (Bhaskar and Hay, 2007; Fayard et al., 2010). In the nucleus, activated AKT phosphorylates and consequently promotes nuclear exclusion and inhibition of FOXO transcription factors, which consist of four family members (FOXO1, FOXO3a, FOXO4, and FOXO6) (Hay, 2011; Ouyang and Li, 2011). Another consequence of AKT kinase activity is activation of mTORC1 via Rheb-GTPase (Huang and Manning, 2009). Several phosphatases negatively regulate the PI3K pathway, including the lipid phosphatases PTEN and SHIP that dephosphorylate PIP3 (Harris et al., 2008), and the protein phosphatase PHLPP that dephosphorylates AKT (Brognard and Newton, 2008). The generation of PIP3 by PI3K also plays a role in the recruitment and activation of other signaling proteins in T cells such as Tec family of kinases (Huang and Sauer, 2010), which have not been extensively studied in T_regs_ and will not be discussed.

## The Role of the PI3K Pathway in T_reg_ Development and Function

The best-defined type of T_regs_ is CD4^+^ and characterized by high and constitutive expression of a transcription factor known as FOXP3. Genetic mutations in FOXP3, which cause defects in the development and function of T_regs_, result in a severe and often fatal multi-organ autoimmune disease called Scurfy in mice and Immunodysregulation, Polyendocrinopathy and Enteropathy, X-linked (IPEX) in humans, illustrating the essential role of Tregs in immune tolerance (Bacchetta et al., 2006; Gambineri et al., 2008; McMurchy et al., 2009, 2010). FOXP3-expressing T_regs_ can be divided into two distinct subsets: natural T_regs_ that develop in the thymus via central tolerance mechanisms, and peripherally induced T_regs_, which differentiate from naïve T cells when self or non-self antigen is encountered in the periphery under tolerogenic conditions. T_regs_ utilize a variety of mechanisms to suppress conventional T cells as well as other immune cells such as macrophages, dendritic cells, and NK cells (Ghiringhelli et al., 2005; Tiemessen et al., 2007; Onishi et al., 2008). Some of the mechanisms used by T_regs_ to suppress immunity include expression of surface inhibitory molecules such as CTLA-4 and CD39, and secretion of anti-inflammatory cytokines such as TGF-β, IL-10, and IL-35 (Vignali et al., 2008; Sakaguchi et al., 2009). This section of the review will discuss the role of PI3K signaling in the development and function of thymically derived natural T_regs_.

### Kinases in the PI3K signaling pathway that affect natural T_regs_

In order to define the function of PI3K in natural T_regs_, most studies have focused on the p110δ catalytic subunit and used mice with a kinase-inactive knocked-in form of p110δ (p110δ^D910A^). The role of p110γ in T_reg_ development and function has not been clearly defined, although chemical inhibition of p110γ can induce peripheral T_reg_ differentiation *in vivo* (Dutra et al., 2011; discussed further below). p110δ^D910A^ mice have an increased proportion of T_regs_ in the thymus, but reduced in the spleen and lymph nodes (Patton et al., 2006). In addition, these T_regs_ are less suppressive and cannot produce the anti-inflammatory cytokine IL-10, as a result, p110δ^D910A^ mice develop spontaneous colitis (Patton et al., 2006) and enhanced resistance to *Leishmania major* infections (Liu et al., 2009). These data suggest that p110δ activity is not required for the development of T_regs_, but rather for their function and maintenance in the periphery. The effect of p110δ inactivation is not specific to T_regs_ since CD4^+^ T cells in these mice are less proliferative and have reduced IL-2, IL-4, and IFN-γ production, suggesting a general impairment in both Th1 and Th2 responses. Despite the defects in T_regs_ and resistance to primary *L. major* infections, p110δ^D910A^ mice are more susceptible to secondary *L. major* infections, due to insufficient generation of Th1-polarized memory cells (Liu and Uzonna, 2010). A subsequent study reported that the p110δ^D910A^ mice have a specific reduction in T_regs_ expressing high levels of CD38, a marker thought to define a highly suppressive population of T_regs_ (Patton et al., 2011). Together these studies suggest that reduced activity of the p110δ form of PI3K is detrimental to the effector and suppressive functions of Th cells and T_regs_, respectively. On the other hand, as discussed below, there is also evidence that excessive activity of PI3K signaling is inhibitory to T_regs_. Thus maintaining the correct threshold of PI3K activity is critical for the normal function of these cells.

Although there is clearly a requirement for a certain level of PI3K activity to maintain T_regs_ in the periphery, T_regs_ have a significantly diminished ability to activate the PI3K pathway downstream of the TCR (Crellin et al., 2007). Diminished signaling is evident not only in terms of reduced AKT phosphorylation, but also at the level of downstream effectors including reduced phosphorylation of p70 S6K and of FOXO1 and FOXO3a at Ser256 (Crellin et al., 2007). Notably, diminished AKT phosphorylation is most evident at Ser473, with normal phosphorylation of Thr308, suggesting that activation of PDK1 is normal. This low activity of AKT is essential for the normal function of T_regs_ since over-expression of an inducibly active form of AKT abolishes their suppressive function (Crellin et al., 2007). Mechanistically, it remains unknown why high activity of AKT block suppression in mature T_regs_ since it does not result in a change in expression of FOXP3, IL-2, CTLA-4, or granzyme B; although trans-differentiation into effector cells may play a role since enforced AKT activation causes T_regs_ to produce high amounts of IFN-γ and IL-4 (Crellin et al., 2007). Constitutive activation of AKT also represses thymic T_reg_ development (Haxhinasto et al., 2008) suggesting that high PI3K activity is detrimental to both the development and function of natural T_regs_.

Many of the studies investigating the role of mTOR in T_regs_ have relied on the use of rapamycin (also known as sirolimus), which selectively inhibits mTORC1 at low doses but can also inhibit mTORC2 at higher doses (Delgoffe et al., 2011). Unlike conventional T cells, T_regs_ are resistant to rapamycin-induced apoptosis (Strauss et al., 2009) and hence this drug can selectively block pro-inflammatory T cells while preserving T_regs_ (Battaglia et al., 2006; Qu et al., 2007; Lu et al., 2010; Zuber et al., 2011) and their suppressive function (Singh et al., 2012). These data support the conclusion that activation of T_regs_ does not require strong activity of the PI3K pathway. Because of this distinct molecular property, the PI3K signaling pathway represents an ideal target for pharmacological immunomodulation. Indeed in mouse models, rapamycin induces T_reg_-mediated tolerance and protects mice against graft rejection (Eng et al., 1991; Zheng et al., 2003; Gagliani et al., 2011), and acute graft versus host disease (Shin et al., 2011). Clinically, use of rapamycin is associated with increased frequency of T_regs_ following lung transplantation (Lange et al., 2010), and increased suppressive activity of T_regs_ in islet transplantation (Monti et al., 2008). On the other hand, some clinical data show an association between rapamycin and an increased incidence of acute rejection (Zuber et al., 2011), possibly due to the parallel ability of rapamycin to expand memory T cells and enhance cytokine production by antigen presenting cells (Saemann et al., 2009; Li et al., 2011). Moreover, rapamycin has many deleterious side effects such as inhibition of islet survival and function (Tanemura et al., 2012), and induction of glucose intolerance and hyperlipidemia (Morrisett et al., 2002; Houde et al., 2010). Thus the favorable effects of rapamycin on immune tolerance must be weighed against the adverse effects of this drug.

### The role FOXO proteins in natural T_regs_

Since natural T_regs_ have diminished AKT activity it was predicted that continued activity of FOXO may be important for their development and function. Indeed, when both FOXO1 and FOXO3a are deleted specifically in T cells, there is reduced development and function of natural T_regs_, resulting in a multi-organ inflammatory disorder (Ouyang et al., 2010). By corollary, enforced FOXO activity results in impaired proliferation and survival of conventional T cells (Fabre et al., 2005), illustrating that the relative activity of this transcription factor is key for maintaining the balance between tolerance and immunity. Mechanistically, FOXO1 and FOXO3a are likely required for T_reg_ development and function because they bind and transactivate the FOXP3 promoter, the essential lineage defining transcription factor for T_regs_ (Ouyang et al., 2010). Interestingly, the FOXO-deficient T_regs_ that do develop produce large amounts of IFN-γ and IL-17, and only weakly express FOXP3, CD25, and CTLA-4 (Ouyang et al., 2010), suggesting that beyond developmental control, FOXO (and the PI3K pathway) can also control the stability of the T_reg_ lineage. Further investigation is required to study how different environments affect the activity of the PI3K in T_regs_ and hence their stability and function.

### The role of phosphatases in the PI3K pathway in natural T_regs_

One reason that could explain why natural T_regs_ have diminished activity of the PI3K pathway could be that they have high activity of one or more of the phosphatases that negatively regulate the pathway. SHIP is a lipid phosphatase that dephosphorylates PIP3 into phosphatidylinositol-3,4-bisphosphate [PI(3,4)P2]. It is now clear that SHIP does not terminate PI3K signaling, but rather modulates it as some proteins, such as TAPP1 and TAPP2, are preferentially recruited to PI(3,4)P2 and initiate distinct signaling pathways (Zhang et al., 2009). SHIP-1^−/−^ mice have an elevated percentage of natural T_regs_ which are suppressive *in vitro* and *in vivo* (Locke et al., 2009), but this apparent enhanced T_reg_ development is likely due to a T cell extrinsic effect of SHIP, since mice with a SHIP-1 deletion only in CD4^+^ T cells do not display this phenotype (Tarasenko et al., 2007). Moreover, T_regs_ do not express high levels of SHIP-1 (our unpublished data), supporting the overall conclusion that there is no intrinsic role for SHIP-1 in T_reg_ development or function.

PTEN is another lipid phosphatase that directly counteracts and terminates the activity of PI3K. T_regs_ from mice with a CD4^+^ T cell specific PTEN deficiency develop and function normally, but they are hyper-proliferative in response to stimulation with IL-2, even in the absence of TCR activation (Walsh et al., 2006; discussed further below). PTEN may thus have an important role in maintaining peripheral T_reg_ expansion by regulating IL-2-induced PI3K signaling in the context of continual expression of the high affinity IL-2R.

Since the reduction of AKT activity in T_regs_ is consistently found at the level of phosphorylation of Ser473 but not Thr308, when a novel Ser473-specific protein phosphatase, known as PHLPP (Gao et al., 2005; Brognard et al., 2007; Liu et al., 2011), was identified in 2005 it was an attractive candidate for a negative regulator of the PI3K pathway in T_regs_. There are two genes in this family: *PHLLP1* and *PHLPP2*. *PHLPP1* is expressed as two isoforms, resulting in a total of three isozymes that differentially control the phosphorylation of the three different isoforms of AKT. We found that both mouse and human natural T_regs_ express significantly more *PHLPP1* mRNA compared to conventional T cells, and moreover, that expression of the protein was critical for their function (Patterson et al., 2011). Although natural T_regs_ in *PHLPP1*^−/−^ mice developed normally, they were dysfunctional both *in vitro* and *in vivo*. In addition, *PHLPP1*^−/−^ T_regs_ had completely restored phosphorylation of AKT at Ser473, suggesting that high expression of PHLPP1 in T_regs_ is the molecular mechanism controlling low activity of the PI3K pathway in these cells. More recent work indicates that PHLPP can also dephosphorylate conventional PKCs, and it will be critical to determine whether T_regs_ also have altered activity of this pathway and how this may impinge on their function (Gao et al., 2005; Brognard et al., 2007; Liu et al., 2011).

## Regulation of PI3K Signaling Pathway by Co-Stimulatory and Co-Inhibitory Molecules in T_regs_

**Figure 2:**
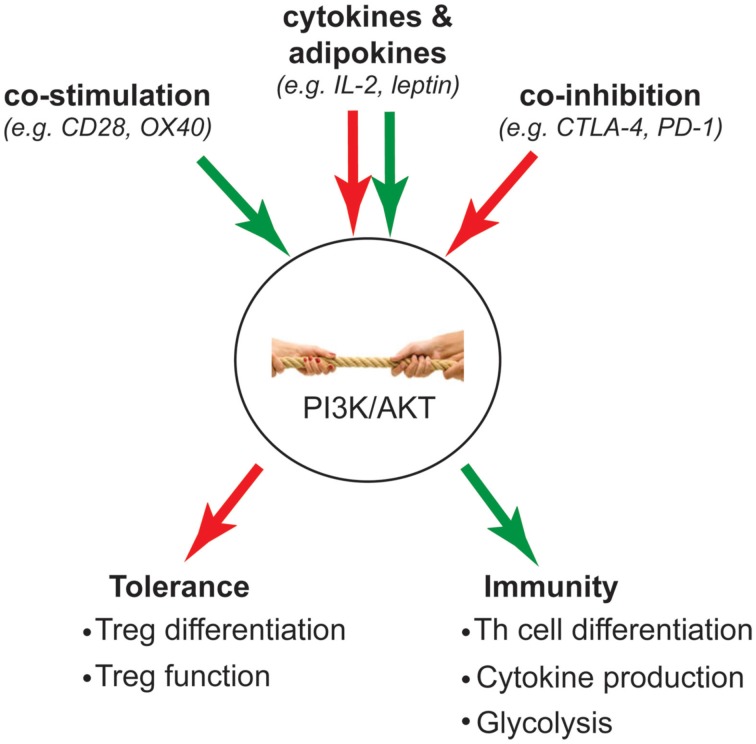
**Intracellular “tug-of-war” determines T cell fate**. Green arrow indicates activation of PI3K signaling; red arrow indicates inhibition of PI3K signaling. The strength of PI3K signaling is influenced by opposing activation and inhibitory signals that are integrated from extracellular stimuli such as co-stimulation, co-inhibition, and cytokines/adipokines. Ultimately, the outcome of this “tug-of-war” determines whether a T cell becomes a T_reg_ to mediate tolerance, or inflammatory Th subsets to mediate immunity.

Much of the biochemistry of the PI3K pathway in T_regs_ has been studied in the context of TCR activation, but it is important consider that this pathway is activated by many different receptors and that the net result will be the integrated signaling that is stimulated by all the factors in the local environment (Figure 2). Co-stimulatory molecules are essential for full T cell activation and have long been known to modulate the level of PI3K signaling induced by the TCR. Indeed CD28-stimulated activation of AKT is a prototypic signaling mechanism that is required for full activation of conventional T cells.

CD28 co-stimulation is an integral part of the development (Tai et al., 2005) and function of T_regs_ (Golovina et al., 2008). Somewhat unexpectedly, we found that even co-stimulation via CD28 in combination with the TCR was not sufficient to restore AKT phosphorylation in T_regs_, illustrating the strength of negative regulation of this pathway in these cells (Crellin et al., 2007; Patterson et al., 2011). In addition to PI3K, many of the effects of CD28 on thymic T_reg_ development are mediated via the NF-κB pathway, and specifically the c-Rel family member (Deenick et al., 2010; Vang et al., 2010). The role of CD28-mediated activation of the NF-κB pathways in fully developed T_regs_ remains to be investigated. In conventional T cells, CD28-mediated activation of the PI3K pathway is necessary for the induction of anti-apoptotic proteins (Okkenhaug et al., 2001), and the induction of glucose uptake via surface expression of GLUT1 glucose transporter (Frauwirth et al., 2002; Jacobs et al., 2008), suggesting that T_regs_, which have diminished CD28-induced PI3K signaling, may use distinct signaling mechanisms to survive and fulfill their metabolic demands (discussed further below). There is evidence that excessive CD28 signaling inhibits immune tolerance, for example, CD28 blockade promotes T_regs_ in organ transplantation (Poirier et al., 2010), but whether the underlying mechanism of CD28 blockade involves modulation of PI3K activity remains to be investigated.

In addition to CD28, the function and biochemical activity of other co-stimulatory and co-inhibitory pathways, such as OX40, CLTA-4, ICOS, and PD-1, have recently been studied in T_regs_. Whereas CLTA-4 and PD-1 suppress PI3K activation, OX40L, and ICOS strongly activate this pathway, leading to the prediction that ligation of the former molecules should promote T_reg_ development and function whereas the latter should block these processes. Curiously, T_regs_ express high levels of all these molecules, suggesting they are poised to have their PI3K pathway turned on or off in response to different environments.

OX40 is expressed on T_regs_ in the absence of immune activation (Sakaguchi, 2004), and, as in activated effector T cells (So et al., 2011), OX40 engagement in T_regs_ activates AKT (Xiao et al., 2012). Studies to investigate whether OX40 engagement positively or negatively affects T_regs_ have produced conflicting data. Some studies suggest that T_regs_ lacking OX40 lose suppressive function *in vivo* (Griseri et al., 2010), while others report that OX40 activation interferes with T_reg_ function (Vu et al., 2007; Piconese et al., 2008). A recent study suggests that the effect of OX40 on T_regs_ may depend on the abundance of IL-2 (Xiao et al., 2012), which activates STAT5 but not the PI3K pathway in T_regs_ (Bensinger et al., 2004). Specifically, OX40 stimulation renders T_regs_ non-suppressive unless IL-2 is abundant. Thus an optimal balance between the PI3K pathway activated by OX40 and the STAT5 pathway activated by IL-2 may be important for regulating both T_reg_ proliferation and function.

ICOS expression defines a subset of effector T_regs_ that are highly suppressive and selectively produce high amounts of IL-10 (Ito et al., 2008) and IL-35 (Whitehead et al., 2012), a phenotype which is likely related to the fact that ICOS expression is induced upon antigen specific activation of T_regs_ *in vivo* (Vocanson et al., 2010). ICOS ligation potently stimulates PI3K activation in conventional T cells (Fos et al., 2008; Simpson et al., 2010), but it is not known whether ICOS stimulation can similarly induce strong PI3K signaling in T_regs_. Thus it remains to be investigated whether the reduced numbers of peripheral T_regs_ in the absence of ICOS (Burmeister et al., 2008) is related to activation of the PI3K pathway in T_regs_.

In contrast to CD28 and other positive co-stimulatory receptors, co-inhibitory receptors such as CTLA-4 and PD-1 typically inhibit TCR-induced PI3K signaling (Parry et al., 2005), and both proteins are highly expressed in T_regs_ (Takahashi et al., 2000; Raimondi et al., 2006). Although CTLA-4 engagement does not inhibit PI3K directly, it is thought that CTLA-4 utilizes the serine/threonine protein phosphatase PP2A to dephosphorylate and inactivate AKT in CD4^+^ T cells (Parry et al., 2005). However, others claim that the inhibitory property of CTLA-4 on T cells is separate from the PI3K/AKT pathway, and that CTLA-4 can signal and activate the PI3K/AKT pathway to promote T cell survival (Schneider et al., 2008). A recent study supports the concept that T_reg_ suppression mediated via CTLA-4 inhibits intracellular signaling in T_regs_ (Tai et al., 2012).

PD-1 stimulation disrupts the accumulation of PIP3 in CD4^+^ T cells by recruiting SHP-2, which subsequently blocks the recruitment and activation of PI3K (Parry et al., 2005; Saunders et al., 2005). PD-L1 and PD-L2 expression on antigen presenting cells, such as tolerogenic dendritic cells, is crucial for efficient differentiation of induced T_regs_ from conventional T cells (Francisco et al., 2009). Mechanistically this role in T_reg_ differentiation is mediated by PD-1-induced down-regulation of AKT and mTOR activity and parallel up-regulation of PTEN (Wang et al., 2007; Francisco et al., 2009; Maldonado and von Andrian, 2010).

Clearly, the effects of these co-receptors on conventional T cells versus T_regs_, and the consequent balance of PI3K signaling are crucial in dictating the state of immune tolerance. As biological agents blocking, or in some cases stimulating, the function of these molecules enter clinical trials (Vincenti, 2007; Weber, 2007; Ford and Larsen, 2009), further research is needed to explore the functional consequences on the activity of the PI3K pathway and the resulting biological effects of T_regs_ versus conventional T cells.

## Regulation of PI3K Signaling Pathway by Cytokines in T_regs_

Cytokines have a major role in directing and sustaining T cell responses, and these molecules also directly regulate the PI3K pathway. Although mature, fully developed T_regs_ respond to many cytokines, to date only the biochemical effects of IL-2 and leptin, an adipo-cytokine, have been intensively studied in these cells. IL-2R signaling is essential for T_reg_ development and survival (Almeida et al., 2002), but the signaling pathway triggered by the receptor is different compared to conventional T cells. Although STAT5 signaling downstream of IL-2R remains intact, as for the TCR, IL-2-stimulated PI3K signaling is selectively inhibited in T_regs_ (Bensinger et al., 2004). This defect in PI3K signaling downstream of the IL-2R has been attributed to the expression of PTEN as PTEN^−/−^ T_regs_ are hyper-proliferative to IL-2 stimulation, even in the absence of TCR stimulation. These data suggest that PTEN is responsible for keeping IL-2-stimulated proliferation of T_regs_ in check despite their continuous expression of the high affinity IL-2R (Walsh et al., 2006). It would be of interest study whether T_regs_ also have defective PI3K pathway activation upon stimulation with other common gamma chain cytokines such as IL-7, which has recently been shown to be required for T_reg_ maturation and homeostasis (Di Caro et al., 2011; Kim et al., 2012), and IL-15, which, much like IL-2, also stimulates expansion of T_regs_ *ex vivo* (Levings et al., 2002). In addition, since polarizing cytokines such as IL-6 and IL-12 have been suggested to affect the stability of the T_reg_ lineage, their downstream receptor signaling pathways should be explored in T_regs_. Finally the neuropeptide hormone vasoactive intestinal peptide inhibits PI3K signaling in T cells and promotes T_reg_ differentiation, indicating that the effects of cytokines which are not normally considered part of the immune response should also be considered (Anderson and Gonzalez-Rey, 2010).

Recent studies have shown that adipocyte-derived cytokines, or adipokines, modulate T cell responses via the PI3K signaling pathway, and that this process affects the function of T_regs_. Most research has focused on leptin, an adipokine induced by food intake and glucose metabolism to control appetite. Specifically, leptin is thought to negatively regulate T_reg_ proliferation by activating mTOR. In parallel, leptin promotes T cell mediated inflammation by enhancing Th1 and Th17 responses, and the survival of autoreactive T cells (Galgani et al., 2010; Matarese et al., 2010). Surprisingly, T_regs_ themselves secrete leptin, and the autocrine effects of this adipokine are thought to induce activation of mTOR (De Rosa et al., 2007; Procaccini et al., 2010). Leptin-induced mTOR activity in T_regs_ causes them to be anergic *in vitro*, and by corollary leptin blockade restores T_reg_ activation and proliferation. Thus oscillatory changes in mTOR activity, controlled partially by leptin, could be necessary for the ability of T_regs_ to vigorously proliferate *in vivo* (Procaccini et al., 2010).

In support of a major role for adipokines in controlling immune tolerance, leptin receptor deficient T_regs_ maintain their suppressive function but have an increased proliferative potential (De Rosa et al., 2007). Similarly, leptin deficient (ob/ob) mice have increased numbers of peripheral T_regs_ and are resistant to experimental autoimmune encephalomyelitis (Matarese et al., 2001). These data contrast to a recent observation that the inflamed adipose tissue in ob/ob mice has a decreased proportion of adipose-resident T_regs_ (Feuerer et al., 2009; Winer et al., 2009), suggesting there may be tissue specific effects of adipokines. Overall, the data from the above studies are consistent with the widely accepted notion that chronic activation of mTOR inhibits T_regs_. With growing evidence that T_regs_ have a role in metabolic disorders, it is important to understand how signals from metabolic and classical immune stimuli are integrated.

## T_regs_ Modulate PI3K Signaling Activity in Target Cells as Part of Their Suppressive Mechanism

Since damping of PI3K signaling is strongly associated with depressed T cell activation, it can be hypothesized that T_regs_ may modulate this pathway in order to suppress their targets. In support of this concept, effector T cells with hyperactive PI3K/AKT activity become resistant to suppression by T_regs_ (King et al., 2006; Ben Ahmed et al., 2009) and T_regs_ attenuate the activation of AKT in CD8^+^ T cells (Kojima et al., 2005). Via CTLA-4 expression, T_regs_ also compete with CD28 expressed on conventional T cells for access to CD80/86 on antigen presenting cells (Greenwald et al., 2005), and can physically remove these co-stimulatory ligands from APCs (Qureshi et al., 2011). As a result, T_regs_ can indirectly limit CD28-induced PI3K activation in their targets. Furthermore, by producing high levels of IL-10, T_regs_ can cause phosphorylation and activation of SHP-1, a tyrosine phosphatase that inhibits the recruitment of PI3K, thus hindering T cell activation (Taylor et al., 2007). In addition, IL-10 can stabilize the expression of SHIP-1 via blocking miR-155, a micro RNA that targets SHIP-1 for degradation, in macrophages (McCoy et al., 2010). Lastly, T_regs_ also express PD-L1 (Francisco et al., 2010), which upon ligation to PD-1 on effector T cells, can inhibit PI3K activity via induction of SHP-2 (Parry et al., 2005). It can be speculated that the ability of T_regs_ to limit PI3K signal strength in conventional T cells would create a condition favorable for peripheral T_reg_ differentiation, hence contributing to infectious tolerance (Kendal et al., 2011).

## The Role of PI3K Signaling Pathway in Peripheral CD4^+^ T Cell Polarization

Depending on the context of stimulation upon activation, naive T cells differentiate into distinct subsets, which are characterized by lineage defining transcription factors and profiles of cytokine production. One arm of T cell differentiation includes the peripheral development of induced T_regs_ which are important for tolerance to harmless commensals and prevention of over-active immune responses against pathogens (Chen and Konkel, 2010). The other arms include Th1, Th2, and Th17 cells, as well as a variety of other newly described Th cell subsets (Zhu and Paul, 2010). Since the relative activity of PI3K plays a key role in regulating Th cell polarization, this in an additional way that the activity of this pathway modulates the balance between tolerance and immunity.

### Kinases in the PI3K Signaling Pathway Which Affect CD4^+^ T Cell Differentiation

Studies involving inhibition of PI3K activity have revealed separate roles for p110δ and p110γ in peripheral CD4^+^ Th polarization. Specific inhibition of p110δ using IC87114 blocks the release of multiple cytokines by human T cells, including IFN-γ, TNF-α, IL-5, and IL-17 (Soond et al., 2010). Similarly, genetic manipulations to inactivate p110δ results in reduced production of IL-4, IL-17, IFN-γ, and IL-10 by different T cell subsets (Okkenhaug et al., 2006; Patton et al., 2006), hence disrupting Th1, Th2, Th17, and T_reg_ associated cytokines. These data suggest that p110δ plays an indispensable role in multiple CD4^+^ Th cell subsets. On the other hand, p110γ does not seem to have a major role in T cell activation (Gruen et al., 2010), and its expression is dispensable for Th1 and Th17 differentiation (Berod et al., 2011). Interestingly, blockade of p110γ by administration of its inhibitor AS605240 in mice can induce T_regs_ *in vivo* and consequently ameliorate colitis (Dutra et al., 2011). Together, these studies suggest that inhibition of p110δ may be beneficial for treating inflammatory disorders where cytokines are over-produced; however, since p110δ activity is essential for T_regs_, immune tolerance would likely not be achieved in parallel. On the contrary, inhibition of p110γ may be beneficial in achieving long lasting tolerance by inducing T_regs_, but may be relatively ineffective at controlling ongoing Th1 and Th17 responses.

There are contradicting results regarding the role of AKT in peripheral differentiation of induced T_regs_. Constitutive AKT activation impairs FOXP3 induction during *in vitro* TGF-β driven T_reg_ differentiation (Haxhinasto et al., 2008), suggesting a requirement for reduced AKT activity in peripheral T_reg_ differentiation similar to that in natural T_reg_ development. In contrast, another study found that in the absence of CD28 co-stimulation, AKT transgenic CD4^+^ T cells have an enhanced capacity to differentiate into T_regs_ (Pierau et al., 2009). In addition, CD28 signaling is required for the survival of induced T_regs_ (Liu et al., 2006), suggesting that in the former study constitutive AKT activity may substitute for the requirement of co-stimulation. On the other hand, CD28 co-stimulation may influence peripheral T_reg_ differentiation via other signaling pathways such as activation of c-Rel, which has been shown to play a role in thymic T_reg_ development (Deenick et al., 2010; Vang et al., 2010). Since AKT is central to various cellular processes including cell survival pathways, it is possible that peripheral T_reg_ development requires some level of AKT activation, provided by CD28 co-stimulation, but which must then be maintained at a relatively low level for the cells to stabilize FOXP3 expression and retain suppressive function.

The activity of mTOR, which forms part of the mTORC1 or mTORC2 kinase complexes when bound to the scaffold proteins Raptor or Rictor, respectively (Laplante and Sabatini, 2009), tightly regulates Th cell differentiation. Deletion of Rictor, which disrupts mTORC2, impairs both Th1 and Th2 differentiation (Lee et al., 2010). The effect on Th1 cells is due to the fact that expression of TBET, the defining transcription factor for Th1 cells, is repressed by FOXO1. In the absence of mTORC2, AKT activity is diminished, FOXO1 is not repressed and hence TBET expression is prevented. In contrast, the effect of mTORC2 deletion on Th2 cells does not seem to be related to AKT or FOXO1. On the contrary, another study reported that while *Rictor*^−/−^ T cells fail to differentiate into Th2 cells, they can still differentiate into Th1 cells (Delgoffe et al., 2011). *Rheb*^−/−^ T cells, which lack the GTPase required for mTORC1 activity, cannot successfully differentiate into Th1 or Th17 cells, but maintain the capacity for Th2 differentiation.

Both mTORC1 and mTORC2 antagonize the peripheral differentiation of T_regs_. While *Rictor*^−/−^ T cells have enhanced TGF-β mediated T_reg_ differentiation (Lee et al., 2010), mTOR-deficient T cells that lack both mTORC1 and mTORC2 readily differentiate into T_regs_ in the absence of TGF-β (Delgoffe et al., 2011). Furthermore, the lack of both mTOR complexes renders T cells unable to skew into Th1, Th2, and Th17 cells (Delgoffe et al., 2009). In agreement with these genetic data, inhibition of mTOR by rapamycin, promotes FOXP3 expression and T_reg_ generation (Kopf et al., 2007; Kang et al., 2008).

In summary, studies of mTOR have shown that mTORC1 is required for differentiation of Th1 and Th17 cells, but not Th2 cells. mTORC2 is most important for Th2 differentiation, but also plays a role in Th1 differentiation, and both mTORC1 and mTORC2 negatively regulate the peripheral differentiation of T_regs_. Hence differential targeting of mTORC1 versus mTORC2 could be used to alter the balance of effector T cell subsets and promote immune suppression.

### The role of FOXO proteins in CD4^+^ T cell differentiation

As discussed above, one of the main ways that the PI3K pathway blocks the differentiation of T_regs_ is via inactivation of FOXO1 and FOXO3a, transcription factors which are necessary for induction of FOXP3 expression (Ouyang et al., 2010). In agreement, ablation of Cbl-b, which results in FOXO3a inactivation in a PI3K dependent manner, also impairs T_reg_ differentiation *in vitro* and *in vivo* (Harada et al., 2010). Impaired T_reg_ differentiation can be rescued by over-expression of FOXO3a, and mice lacking FOXO3a have increased Th1 and Th2 cells (Lin et al., 2004). Together these data indicate that regulation of FOXO activity is the critical arm of the PI3K pathway controlling the balance between immune tolerance and inflammation.

### Phosphatases in the PI3K signaling pathway that affect CD4^+^ T cell differentiation

As negative regulators of the PI3K pathway, phosphatases such as SHIP also have crucial roles in Th cell differentiation. Systemic *SHIP-1* deletion results in reduced numbers of Th17 but not Th1 cells. Furthermore, when naive T cells from *SHIP-1*^−/−^ mice are transferred into immunodeficient mice, they are less able to induce colitis, possibly due to their reduced IL-17 production and parallel tendency to differentiate into induced T_regs_ (Locke et al., 2009). In studies of mice with a T cell specific *SHIP-1* deletion, *SHIP-1*^−/−^ T cells themselves have a reduced capacity to differentiate into Th2 cells (Tarasenko et al., 2007). Furthermore, *SHIP-1*^−/−^ Th2 cells produce less IL-4, suggesting that SHIP-1 is an intrinsic positive regulator of Th2 responses (Tarasenko et al., 2007; Roongapinun et al., 2010). Interpretations on effects of the PI3K pathway from these studies of *SHIP*^−/−^ T cells have to be taken with caution as SHIP does not simply reverse PI3K activity, but rather modulates the downstream signaling effects through a modified lipid second messenger PI(3,4)P2, which can also act by recruiting adaptor proteins (Zhang et al., 2009).

T cell deficiency of *PTEN* results in enhanced AKT activation and resistance to TGF-β driven differentiation of induced T_regs_ (Sauer et al., 2008). On the other hand, T cell specific *PTEN* deficiency also causes uncontrolled proliferation and cytokine production by both Th1 and Th2 cells, ultimately leading to the development of lymphoma (Suzuki et al., 2001). Collectively, PTEN is necessary to keep T cell proliferation in check and maintain tolerance. We have shown that expression of PHLPP is crucial for the induction of FOXP3 expression in T cells (Patterson et al., 2011). When *PHLPP1* is deleted, conventional T cells lose the ability to convert into induced T_regs_ in the presence of TGF-β. Furthermore, PHLPP expression is up-regulated in response to TGF-β, consistent with high PHLPP expression found in natural T_reg_.

Overall, the differentiation of Th cells into distinct subsets is clearly modulated by the PI3K pathway. Since these different Th cell subsets have distinct roles in different immune responses, modulating the pathway could be used in different therapeutic approaches. For example, in the case of infectious diseases, it may be advantageous to enhance PI3K activity and block T_regs_ and Th2 cells. On the other hand, since inhibitors of p110α, p110γ, AKT, or mTOR all favor the conversion of conventional T cells into T_regs_ (Fruman and Bismuth, 2009; Dutra et al., 2011), these agents have promise in strategies to induce tolerance.

## The Role of PI3K Signaling Pathway in Regulating T Cell Metabolism

Cellular metabolism is a previously under-studied aspect of T cell biology that has recently gained much attention. As with all cells, T cells have energy requirements and must generate ATP to survive and function. In their naive quiescent state, T cells rely on oxidative metabolism to survive. Upon activation, however, T cells increase their energy requirements to support proliferation and effector functions such as cytokine production. Activated effector T cells must meet this increase of demand for energy and building blocks for cellular macromolecules by switching to the catabolic process of glycolysis (Maciver et al., 2008).

Upon TCR activation in conjunction with CD28 co-stimulation, T cells increase their ability to uptake glucose by promoting surface trafficking of the glucose transporter GLUT1 and glycolysis via a process that depends on the PI3K signaling pathway (Frauwirth et al., 2002; Jacobs et al., 2008). If co-stimulation is lacking, T cells have a reduced ability to proliferate due to failure to activate PI3K and increase glycolysis. Furthermore, T cells with constitutive AKT activation have increased glycolytic activity, and lose their dependence on CD28 co-stimulation to proliferate and secrete cytokines. Since ICOS and OX40 co-stimulatory molecules induce strong PI3K activity on activated T cells, it is possible that their stimulation promotes even stronger glycolytic activity on antigen experienced T cells. In line with this observation, activation of co-inhibitory receptors CTLA-4 and PD-1, and the use of inhibitors of the PI3K pathway, prevents the up-regulation of glucose uptake in T cells (Frauwirth et al., 2002; Parry et al., 2005; Wieman et al., 2007). In this section, we will review the differential cellular metabolic requirements between T_reg_ and conventional T cells as they relate to the PI3K signaling pathway.

The distinct lineages of CD4^+^ Th cells differ in their metabolic requirements. Even though Th1, Th2, and Th17 cells all express GLUT1 and require glycolysis (Michalek et al., 2011), Th17 cells uniquely require a protein known as HIF-1α for their glycolytic activity (Shi et al., 2011). Expression of HIF-1α in Th17 cells requires mTOR activation, and thus inhibition of mTOR by rapamycin blocks HIF-1α induction and expression of glycolytic enzymes in Th17 cells. HIF-1α is a transcription factor which responds to changes in oxygen tension and directs cells to switch from oxidative phosphorylation to aerobic glycolysis (Semenza, 2007). Indeed hypoxia, which activates HIF-1α, promotes skewing toward Th17 cells and away from T_regs_ (Dang et al., 2011). Similarly, *HIF-1*α^−/−^ T cells have defective Th17 differentiation, and are more prone to express FOXP3 and become T_regs_. Interestingly, HIF-1α has been reported to bind and target FOXP3 for ubiquitination and proteasomal degradation (Dang et al., 2011), providing a possible mechanism for the observed effects on T_regs_. Along with the role of FOXO on FOXP3 expression and T_reg_ function, these recent findings on HIF-1α provide an additional mechanism for how activation of the PI3K pathway can negatively regulate T_regs_.

Unlike Th1, Th2, and Th17 cell subsets, T_regs_ and memory T cells are relatively quiescent, expressing low amounts of GLUT1 and not requiring high glycolytic activity (Michalek et al., 2011). Instead of glycolysis, T_regs_ depend on AMPK, an enzyme which antagonizes mTOR activation, to perform lipid oxidation and meet their energetic demands. Metformin, a drug commonly used as to treat type 2 diabetes, activates AMP, and increases lipid oxidation and T_reg_ numbers *in vivo* (Michalek et al., 2011). Since enhancing T_reg_ numbers *in vivo* ameliorates insulin resistance in mice (Feuerer et al., 2009; Winer et al., 2009), further investigation into whether part of the mechanism of action of metformin in type 2 diabetes is related to enhanced T_reg_ function is warranted.

Since AMPK inhibits Rheb-GTPase mediated mTORC1 activation (Laplante and Sabatini, 2009), modulating the balance between mTOR and AMPK can be used to alter T cell metabolism and hence lineage differentiation. For example, rapamycin-mediated inhibition of mTOR favors AMPK activity and the lipid oxidation of T_regs_ (Michalek et al., 2011). Rapamycin can also reverse the effect of *AMPK* or *LKB1* (upstream kinase for AMPK activation) deletion, resulting in increased mTORC1 activity, glycolysis, and over-production of IFN-γ (Maclver et al., 2011). Since T_regs_ and memory T cells are metabolically similar, it is no surprise that rapamycin can promote the generation of both of these cell types (Araki et al., 2009; Golovina et al., 2011). Interestingly, TCR stimulation can activate both mTOR and AMPK (Tamas et al., 2006), and therefore, the relative strength of the PI3K pathway activation may be crucial in determining whether a T cell passes the threshold of mTOR activity to proceed to glycolysis.

Notably, one of the mechanisms that T_regs_ use to suppress conventional T cells is through metabolic disruption via CD39, an ectonucleotidase that hydrolyzes extracellular ATP (Borsellino et al., 2007). AMPK is preferentially activated in conditions of high AMP:ATP ratio (Carling et al., 2011). Thus via CD39, T_regs_ may be able to promote AMPK activity in their target cells, ultimately antagonizing mTOR activity. AICAR, a drug that promotes the activation of AMPK, has been shown to promote T cell anergy (Zheng et al., 2009), supporting the notion that AMPK activity is beneficial for immune tolerance.

## Conclusion

Collectively, the above studies reveal the complexity and intricacies of signaling requirements for T_regs_ and different Th cell subsets. The studies of mice expressing p110δ^D910A^ reveal that too little activity of the PI3K/AKT pathway is detrimental for T_regs_. On the other hand, many studies show that strong PI3K/AKT signaling activity negatively affects T_regs_. These differential effects suggest that there is likely a certain range of PI3K/AKT signal strength that is permissive for T_regs_. This signal strength is likely determined by the collective outcome of various extracellular stimuli that can activate or inhibit PI3K/Akt signaling, thus regulating cellular changes (Figure 2). Because the PI3K/Akt pathway serves as a critical signaling hub, which directs the balance between inflammation and immune tolerance, it is an ideal target for therapeutic manipulation.

## Conflict of Interest Statement

The authors declare that the research was conducted in the absence of any commercial or financial relationships that could be construed as a potential conflict of interest.
